# Clinical Value of Serum S100A8/A9 and CA15-3 in the Diagnosis of Breast Cancer

**DOI:** 10.30699/IJP.14.2.104

**Published:** 2019-06-10

**Authors:** Samaneh Khorrami, Masoumeh Tavakoli, Elahe Safari

**Affiliations:** 1 *Immunology Research Center, Iran University of Medical Sciences, Tehran, Iran*; 2 *Department of Immunology, School of Medicine, Iran University of Medical Sciences, Tehran, Iran *; 3 *Department of Biochemistry, School of Medicine, Iran University of Medical Sciences, Tehran, Iran*

**Keywords:** Breast cancer, Tumor marker, Serum, Enzyme-linked, immunosorbent assay

## Abstract

**Background and Objective:**

S100A8/A9 is a heterodimer calcium-binding protein which is involved in tumor cell proliferation, adhesion and invasion, and is proposed as a biomarker for better diagnosis and prognosis in many cancers. The aim of this study was to evaluate the simultaneous serum-based level of S100A8/A9 and CA15-3 as well-illustrated cancer biomarkers, as well as their prognostic value in breast cancer patients and healthy matched controls.

**Material and Methods:**

Thirty breast cancer patients at different stages of disease and healthy matched controls with no history of inflammatory, autoimmune diseases, or cancer, were enrolled in the study. The levels of S100A8/A9 and CA15-3 were assessed serologically using the Enzyme-linked immunosorbent assay (ELISA) method, and the relevance of these markers with patients’ clinicopathological features were subsequently assessed.

**Results:**

Based on our data, the serum levels of both S100A8/A9 and CA15-3 were significantly higher in patients compared to the healthy controls, and thus positively correlated with tumor size. Also, statistical analysis shows that the serum level of S100A8/A9 has 100% specificity and sensitivity (AUC = 1.00, 95% CI) for the diagnosis of breast cancer patients.

**Conclusion:**

According to our data as well as other observations, the S100A8/A9 heterodimer can be considered as a potential biomarker for the proper diagnosis and prognosis of breast cancer.

## Introduction

Breast cancer is one of the leading causes of cancer-related mortality and morbidity among women globally, and it poses a notable metastatic-induced death subsequently. The most used therapeutic approaches for breast cancer are hormone-dependent and chemotropic-based strategies that lack the efficiency to meet the requirements of patients’ feature spectrums and patient outcomes (1). Therefore, finding sensitive, specific and reliable biomarkers for earlier diagnosis and post-operative follow up of patients is a priority for many research groups. A variety of biomarkers is proposed to consider as an indicator of normal, pathologic conditions and response to different treatments, however finding the specific biomarkers which benefit from being noninvasive, easy to reach and affordable, has yet to be elucidated (2). Cancer antigen 15-3 (CA15-3) known as MUC-1 is a trans-membrane glycoprotein lining the surface of epithelial tissue, providing further protection (3). It has been shown that its positive effect in cancer formation is through its interaction with intracellular signaling pathways that control proliferation, and with adhesion molecules such as ICAM, which suggests that it is an effective protein with invasive and metastatic properties (4, 5). Multiple lines of evidences revealed that CA15-3 is elevated in the serum of patients suffering from breast cancer, and suggested it as a useful and non-invasive biomarker for the prognosis, diagnosis and monitoring of breast cancer, however it failed to detect patients at early stages of localized breast cancer (3, 6). 

S100A8/9 or Calprotectin is a heterodimer protein belonging to a family of calcium-binding proteins, which is abundant in neutrophils and secreted by activated immune cells (7). It has been shown that S100A8/9 acts as a danger signal (Damage Associated Molecular Pattern molecule or DAMP) and following release to the extracellular space can stimulate and recruit immune cells and induce an immune response (8). The relevance of Calprotectin to carcinogenesis has been well-described recently and its up-regulation is reported in many cancers including colon, lung and gastrointestinal. It has been proposed that the involvement of S100A8/9 in triggering inflammatory pathways and cell cycle regulation is the key reason that connects S100A8/9 to carcinogenesis mediated by inflammation. However, its down-expression was reported in cells from head and neck squamous cell carcinoma (HNSCC) and its lower expression was accompanied by a higher proliferation and more invasive phenotype of tumor cells (9). Interestingly, the activity of the S100A8/9 heterodimer is in a tissue-dependent manner also related to protein localization, since the extra and intracellular localization of S100A8/9 can affect its properties and function. Extracellular S100A8/9, which is secreted via infiltrated immune cells or epithelial cells is involved in inflammatory-dependent tumor cell progression and development (9, 10), therefore it can serve as a promising circulating biomarker for cancer diagnosis (11). Bansal et al, have shown that the level of S100A8 and 9 proteins was significantly reduced post-operatively in patients with bladder cancer (12). Based on recent evidence, S100A8/9 can influence cancer cell growth and proliferation through the apoptotic pathway in a dose-dependent manner mediating the MAPK and NF-κB signaling pathways (9). In light of such studies, the prognostic value of S100A8/9 as a multi-functional protein is yet to be determined, and it provoked us in the present study to evaluate the circulating level of the S100A8/9 heterodimer as a proposed biomarker and CA15-3 as a well-described biomarker in patients with different stages of breast cancer against healthy-matched controls in order to assess the prognostic value of these markers and determine their correlation to the clinicopathological features of patients. 

## Materials and Methods

**Patients and sample collection**

In the present study, 30 patients with ductal carcinoma and 24 healthy controls with local ethical approval and informed consent were enrolled in the investigation. Patients were referred to the Iran National Tumor Bank, at Imam Khomeini Hospital, Tehran, Iran. Healthy controls who did not have a family history of cancer, any history of inflammation or immune-related diseases were included in the study. Also, there was a confidence that patients did not receive anticancer therapy and were diagnosed to have breast cancer (stage I-III) histopathologically. The patients’ clinical pathological features were taken from the Iran National Tumor Bank, Imam Khomeini Hospital, Tehran, Iran and histopathologic features were confirmed by immunohistochemistry. 

**Blood collection, processing and storage**

In order to prepare samples for experiments, blood samples were taken from patients and healthy controls. Following centrifugation at 1200 g for 10 minutes, the serum was separated from each sample and stored at -80 ºC until use. 

**Measurement of S100A8/A9**

In order to evaluate the level of S100A8/A9 in the serum samples of patients and controls, Enzyme-linked immunosorbent assay (ELISA) was applied according to the manufacturer’s instructions (Biovendor, brno, Czech Republic, USA). Briefly, 100 µl of diluted samples were added in ELISA plate wells which were coated with polyclonal anti-human S100A9 antibody. Following proper incubation and washing, HRP-labelled second polyclonal anti-human antibody was added and incubated with captured S100A8/A9 for 60 minutes. After further washing, substrate solution was added, then the reaction was stopped using an acidic solution and the absorbance of all wells was measured using a microplate reader (Bio-Rad) at 405 nm. The detection limitation of the S100A8/A9 ELISA kit was 0.22 ng/ml and samples were double checked.

**Measurement of CA15-3:**

In order to evaluate the level of CA15-3 in the serum sample of patients and controls, the ELISA method was applied using the manufacturer’s instructions (CanAg CEA EIA kit, Fujirebio, USA). Briefly, samples were added into ELISA plate wells coated with CA15-3-immobilized antibody and following adequate incubation and washing, wells were exposed with biotinylated anti-human antibody and washed again. Subsequently, HRP-conjugated streptavidin was added to the wells and following proper incubation and washing steps, the reaction in the wells was stopped and the absorbance measured at 405 nm by microplate reader (Bio-Rad). The detection limit was <1 ng/mL.

**Statistical analysis:**

To evaluate and compare the circulating level of S100A8/A9 and CA15-3 in patients and control groups, statistical tests including t test, one-way ANOVA and post hoc Dunnett test were applied. The observed differences were considered significant when P value was less than 5% or (*P*<0.05) and statistically significant differences were shown in the figures using asterisks. The statistical analyses were done using Graph Pad Prism statistical software and SPSS software. Also, Receiver operator characteristic (ROC) curves and the areas under the curve (AUC) with 95 % confidence interval (CI) were calculated using Graph Pad Prism software. The sensitivity and specificity were analyzed to evaluate the diagnostic performance of S100A8/A9.

## Results

Clinicopathological characteristics of patients:

In the present study 30 patients suffering from breast cancer and 24 healthy-match controls were enrolled in the investigation, the clinic-pathological features of whom are summarized in Table 1. Briefly, the average age of breast cancer patients was 48.1 years old and their distribution as a point of stage was 2 (6.6%) at stage I, 20 (66.6%) at stage II and 8 (26.6%) at stage III. Additionally, 17 (56.6%) cases were ER (estrogen receptor) positive and 11 (36.6%) cases were negative. Also, 21 cases (70%) were negative for Her2 while 7 (23.3%) were positive. The number of patients with pathological features of lymphatic and vascular invasion were almost the same, as the number of lymphatic-invasion positive and negative cases was 13 for both (43.3%) and the number of vascular-invasion positive and negative cases were 14 (46.6%) and 12 (40%), respectively. As for the type of tumor, 27 cases (90%) were diagnosed for invasive ductal carcinoma, 2 (6.6%) for DCIS, and 1 (3.3%) for *in situ* and invasive ductal carcinoma

**Table 1:** Clinicopathological Features of Patients

Parameters	Groups	Numbers
Age	20-40 40-60 ≥60	9 16 5
TNM Stage	I II III	2 8 17
Grade	I II III	6 9 15
Lymphatic Invasion	Yes No	13 13
Vascular Invasion	Yes No	14 12
Tumor Size	≤2.5 cm >2.5 cm	9 21
Her2 status	Positive Negative	7 21
ER status	Positive Negative	17 11
PR status	Positive Negative	16 12
P53 status	Positive Negative	12 6

ER: Estrogen receptor, PR: Progestogen Receptor

**The circulating level of S100A8/9:**

**Figure 1:**
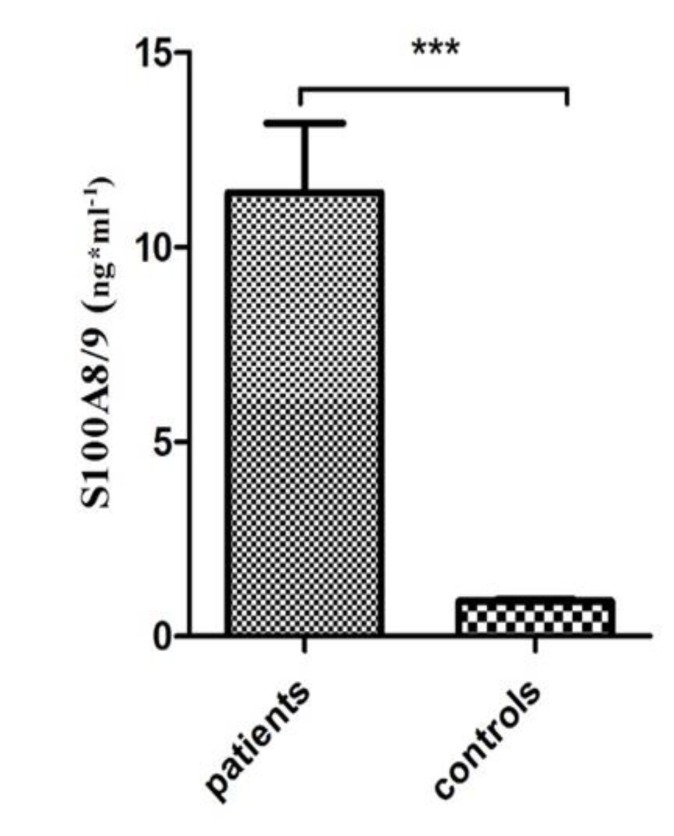
The serum level of S100A8/9 in patients and controls. The serum level of S100A8/9 was higher in patients compared to controls (*= P <0.05, **= P <0.01, ***= P <0.001).

**Figure 2:**
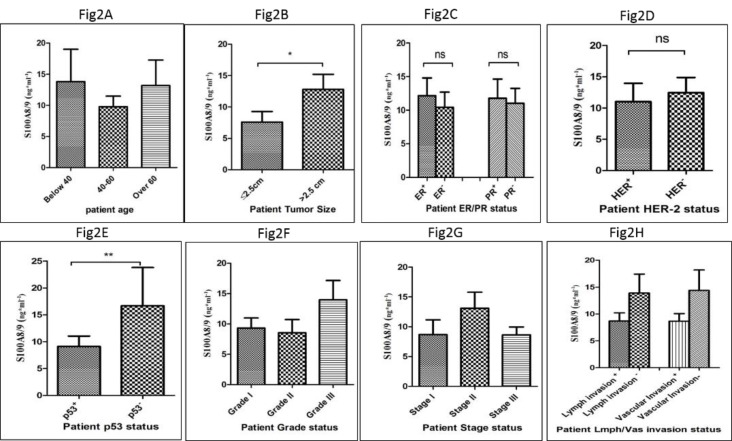
The correlation of the level of S100A8/9 with patients’ clinicopathological features is shown according to patient age distribution (A), tumor size (B), ER and PR statues (C), Her2 status (D), P53 status (E), Grade of cancer (F), Stages of cancer (G) and lymph/vascular invasion (H). (*= P <0.05, **= P <0.01, ***= P <0.001)

The serum level of the S100A8/9 heterodimer was assessed using the ELISA method in patients and controls according to the manufacturer’s instructions. According to the results, the level of S100A8/9 was significantly higher in patients compared to healthy controls *(P<0.0001)* as shown in Fig 1. However, the level of circulating S100A8/9 showed no significant differences regarding patients’ age distribution (Fig 2A). A positive correlation was noted in the level of S100A8/9 in patients with a large tumor size (>2.5 cm) compared to a small tumor size (≤2.5 cm) (*P<0.01*) (Fig 2B). Despite the higher level of S100A8/9 in the ER positive and PR positive patient group, the difference was not statistically significant compared to the negative group (Fig 2C). Also, despite the marginally higher level of S100A8/9 in the Her2 negative group compared to the Her2 positive group, no significant correlation was observed subsequently (Fig 2D). The level of S100A8/9 in the p53 negative group was significantly higher comparing to the p53 positive group *(P<0. 001) *(Fig 2E)*. *Moreover, a higher level of S100A8/9 in patients at grade III was observed, however no positive correlation was observed compared to the level of this protein in patients at lower grades (Fig 2F). Similarly, no positive correlation was noted in the level of S100A8/9 among patients at different stages, however patients at stage II showed a higher level of S100A8/9 (Fig 2G). Despite the higher level of S100A8/9 observed in patients who were negative for lymph and vascular invasion, this difference, compared to the positive group, was not statistically significant (Fig 2H). 

**The circulating level of CA15-3:**

**Figure 3:**
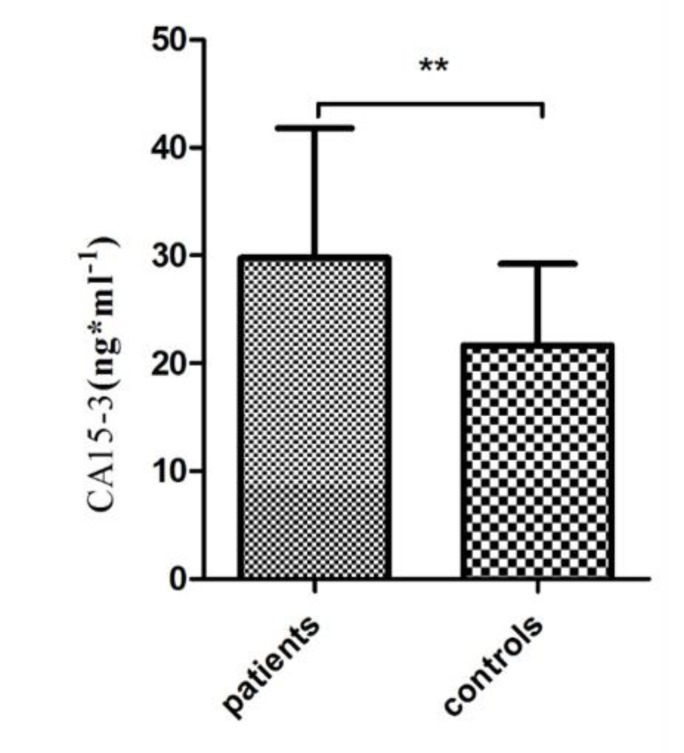
The serum level of CA15-3 in patients and controls. The serum level of CA15-3 was higher in patients compared to controls (*= P <0.05, **= P <0.01, ***= P <0.001)

**Figure 4:**
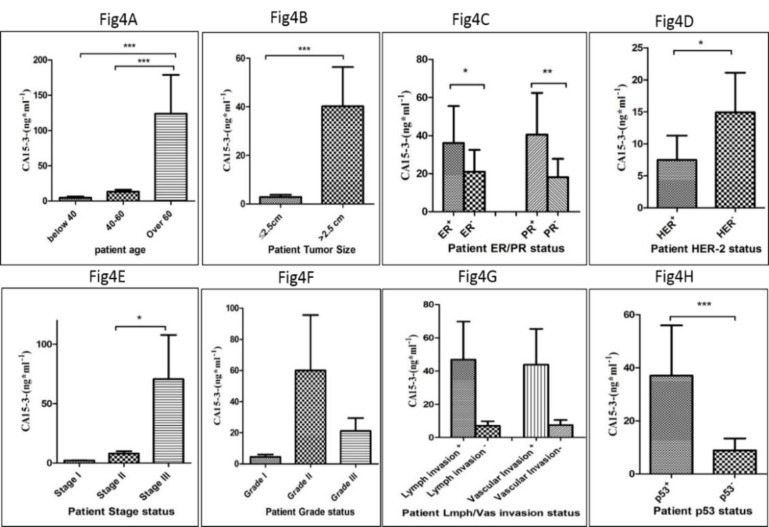
The correlation of the level of CA15-3 with patients’ clinicopathological features is shown according to the patient age distribution (A), tumor size (B), ER and PR statues (C), Her2 status (D), P53 status (E), grade of cancer (F), stages of cancer (G) and lymph/vascular invasion (H) (*= P <0.05, **= P <0.01, ***= P <0.001)

In order to determine the level of CA15-3 in the serum of patients suffering from breast cancer and healthy matched controls, the circulating level of CA15-3 was assessed using the ELISA method based on the manufacturer's instruction. Based on the results presented in Fig 3, the serum level of CA15-3 in patients was significantly higher in comparison to the healthy group *(P*<0. 001*).* In addition, the level of CA15-3 showed a positive correlation in patient age since patients over 60 years of age showed a remarkably higher level of CA15-3 compared to other age groups (*P*<0. 0001) (Fig 4A). This difference in the serum level of CA15-3 was also observed in patients whose tumor size was larger than 2.5 cm compared to patients with a smaller tumor size (*P*<0. 0001) (Fig 4B). Also, a positive correlation was observed in the level of CA15-3 in ER-positive (*P*<0. 01) and PR-positive (*P*<0. 001) patients, compared to the negative groups (Fig 4C). Notably, there were significant differences in the serum level of CA15-3 among patients who were negative for Her2 compared to the Her2-positive group (*P* <0. 01) (Fig 4D). A significant difference was also observed in the level CA15-3 in the serum of patients who were positive for p53 compared to the p53-negative group (P<0. 0001) (Fig 4E). A significant correlation was observed in the level of CA15-3 in patients at stage III of the disease vis-a-vis other stages (*P* <0. 01) (Fig 4G) however the difference in the level of CA15-3 among different grades were not statistically significant (Fig 4F). Despite of higher level of CA15-3 in patient groups with proven lymphatic and vascular invasion, the observed differences were not significant (Fig 4H).

**Potential diagnostic values of CA15-3 and S100A8/A9**

**Figure 5:**
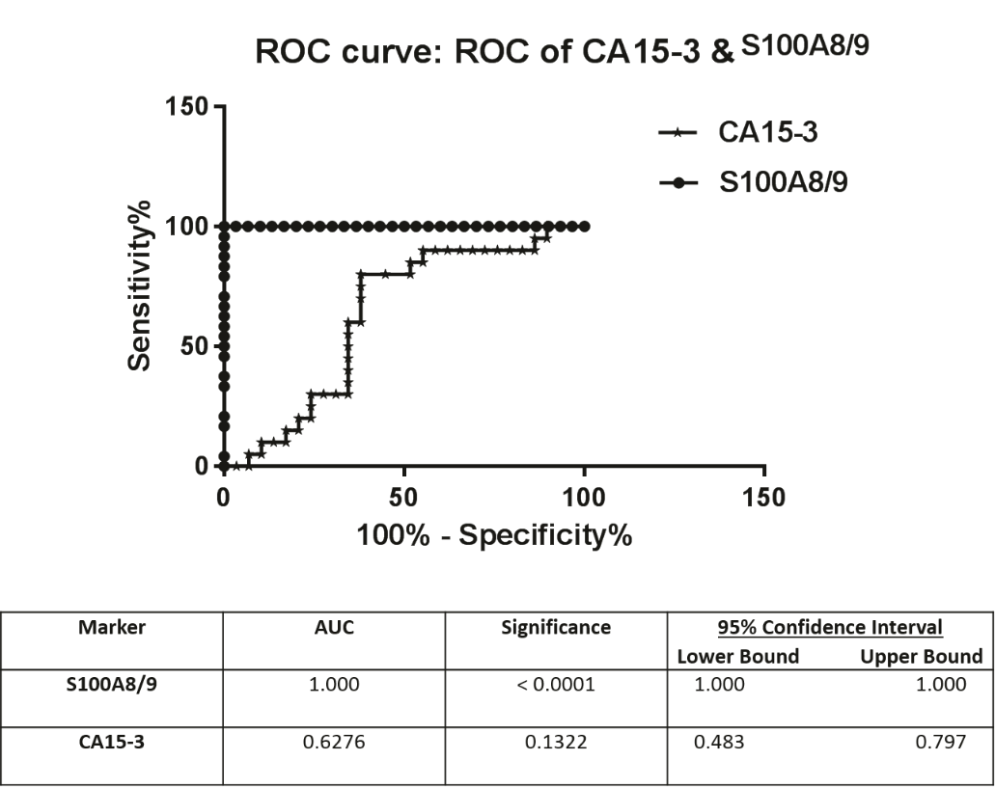
ROC plot and AUC for S100A8/9 and CA15-3 for patients and controls

The receiver operating characteristic (ROC) plot and AUC for CA15-3 and S100A8/A9 were performed (Fig 5). The AUC for S100A8/A9 was remarkably high (1.00) and, as presented, the distinction can be clearly seen between patients and healthy individuals (*P* <0.001) but the AUC for CA15-3 was 0.62 and *P* =0.13, and thus not statistically significant. Sensitivity and specificity values for S100A8/A9 were calculated and presented in Table 2. The sensitivity and specificity of S100A8/A9 were notably high (100%). 

**Table 2:** Sensitivity, specificity, PPV and NPV of markers in patients and controls

Marker	Sensitivity %	Specificity %
**S100A8/9**	100	100

## Discussion

Breast cancer is one of the most life-threatening diseases among women, and is a heavy burden for a patient to hold. Efforts are in progress to delineate the molecular mechanism underlying breast cancer initiation, development and invasion (13, 14). The lack of efficient prognostic and diagnostic markers for the detection and follow up of breast cancer, and the requirement of reaching more profitable therapeutic approaches is at the core of many scientific efforts recently. In the present study, we aimed to evaluate and compare the prognostic value of the S100A8/9 heterodimer, whose biomarker potentiality has been gaining prominence as of late, and CA15-3 as a more familiar cancer biomarker in breast cancer to further provide evidence for the serum-based applicability of these proposed markers. S100A8/9 proteins are calcium-binding heterodimers that are produced and secreted by immune cells, like neutrophils, and involved in inflammatory responses (11). It has been demonstrated that S100A8/9 obeys the tissue-based pattern in cancer progression, and its up regulation was reported in colon, lung and gastrointestinal cancers; however its down-regulation was reported in head and neck squamous cell carcinoma (9, 15). The serological level of S100A8 and 9 proteins were decreased post-operatively in bladder cancer (12). Our results have shown that the serum level of S100A8/9 was significantly higher in patients vis-a-vis healthy controls, and this over-expression was positively correlated with tumor size, which is in line with the reported higher gene expression of S100A8 and S100A9 in breast cancer (16). Our data revealed a slightly higher level of S100A8/9 in ER- and PR-positive and Her2-negative patient group; however, these observed differences were not statistically significant. It has been shown that ER-positive breast cancer cases have a better prognosis compared to ER-negative cases, which form the majority of breast cancer patients.

Our data showed that S100A8/A9 values have substantial differences between patients and controls, and showed the highest AUC (1.00). Also, according to our data, the sensitivity and specificity of S100A8/A9 were very high (100%). But the CA15-3-related ROC-AUC value of our study was 0.62 and was not significant, which might be due to the fact that the CA15-3 level can be affected by other factors including the inflammatory, genetic and physiological background of individuals.

The responsiveness of Her2-positive breast cancer patients to therapy is low, much like their survival rate (16). Yin et al, have shown that S100A8/9 controls breast cancer cell growth and invasion through its surface receptor (17). More recently it has been shown that the expression level of S100A8/9 in Her2-positive breast cancer patients was increased and the level of ER was decreased following the administration of S100A8/9 in the MCF-7 cell line (16). Also, Acharyya S et al, have shown the importance of S100A8/9 with TNF-α as paracrine mediators of the tumor environment, which facilitate breast cancer cell survival (18). Rodriguez-Bathe rrueco et al, demonstrated that S100A8/9 is induced following the activation of the STAT3 pathway, and that it promotes the proliferation of HER2-negative/HER2-positive breast cancer cells (19). Li et al, have shown that the low concentration range of S100A8 and 9, each and together enhance the proliferation, migration and vessel formation of the human umbilical vascular endothelial cell (HUVEC) line. Thus these proteins, apart from their original sources, can stimulate angiogenesis and tumor development (20). 

It has been shown that the inhibition of S100A8 and 9 in cancer cells resulted in abrogated tumor cell invasion and migration, and led to the subsequent down-regulation of matrix-metalloproteinase (MMP) 2, 9 which further emphasizes the implication of S100A8 and 9 in tumor invasion (8). However, our results have not shown a higher level of S100A8/9 in patients with a poor prognosis of breast cancer (ER-negative/Her2-positive); also, this higher level was not observed in the lymph/vascular invasion positive group. This might be explained by the low number of patients involved in this study, and that most of the patients who enrolled in the study were ER-positive and Her2-negative. Therefore, the survey should be expanded by including more patients from diverse breast cancer sub-groups. It should be noted that the serological level of the S100A8/A9 heterodimer was assessed in our study, while more previous studies were focused on measuring S100A8 and S100A9 separately. 

Moreover, the level of CA15-3 was significantly higher in breast cancer patients compared to healthy-matched controls based on our data, and this high level positively correlated with patients’ ages and tumor sizes, as well as the presence of p53 and lymph and vascular invasion. Our data is in line with evidence on the relevance of CA15-3 with breast cancer progression and development (21) and that this protein can be considered as a prognostic biomarker in breast cancer (5, 6).

Taken together, the higher level of circulating S100A8/A9 in the serum of breast cancer patients and the simultaneous elevation of CA15-3 might provide evidence for supporting the potential role of the S100A8/A9 heterodimer and CA15-3 in the prognosis, diagnosis and monitoring of breast cancer, although according to statistical analysis and the Roc Curve Plot S100A8/A9 probably is much better than CA15-3 because the results show that S100A8/A9 has 100% sensitivity and specificity for the diagnosis of breast cancer patients. Little studies have been devoted to assess and compare the prognostic value of these markers and the result of our study attempted to provide such important data, however more expanded investigations are required to evaluate breast cancer biomarkers more comprehensively.
